# Deconstructability prediction for building using machine learning and ensemble feature selection techniques

**DOI:** 10.1038/s41598-025-00790-0

**Published:** 2025-07-01

**Authors:** Habeeb Balogun, Hafiz Alaka, Eren Demir, Christian Nnaemeka Egwim, Godoyon Ebenezer Wusu, Wasiu Yusuf, Muideen Adegoke, Iqbal Qasim

**Affiliations:** 1 Big Data Technologies and Innovation Lab., University of Hertfordshire, Hatfield, United Kingdom; 2 School of Computer Science and Engineering, University of Westminster, London, United Kingdom

**Keywords:** Civil engineering, Applied mathematics, Computer science, Environmental impact

## Abstract

Construction industries remain one of the most significant users of materials and generators of waste in the UK and globally. Notwithstanding, the principle of circular economy is becoming prominent as an effective means for powering greater resource efficiency. It has the prospect of unlocking significant economic value, particularly at the building end of useful life through reuse. A noteworthy end-of-life practice which aligns with this idea is deconstruction, which is the careful disassembly of the building into components and sub-components for reuse. However, deconstruction is not meant for all buildings, and this is because a typical building is constructed as a permanent product waiting to be disposed of after use. Laying on this foundation, assessing the building for deconstruction is necessary, and it is mainly done via several manual inspections, which may be expensive and time-consuming. A deconstructability predictive model using a machine learning-based model and ensemble feature selection techniques was developed to tackle this problem. This paper elaborates on the model creation and illustrates its application through a real-world deconstruction project.

**Supplementary Information:**

The online version contains supplementary material available at 10.1038/s41598-025-00790-0.

## Introduction

The construction sector generates an immense volume of waste annually, with demolition activities contributing significantly to this problem^1^. Construction and demolition waste (C&D) constitutes 30–40% of global waste, exceeding 5 billion tons annually^2,3^. Among major contributors, China produces over 2.3 billion tons of waste, the USA over 500 million tons, and the UK over 100 million tons^4^. Much of this waste arises from buildings demolished after an average lifespan of 30 years or due to abandonment or natural disasters^5^. Notably, demolition transforms more than 80% of a building into waste^6^.

Deconstruction offers a sustainable alternative to demolition by enabling the recovery and reuse of building materials and components. Unlike demolition, which is destructive, deconstruction involves deliberate dismantling to maximise material recovery^6^. Studies indicate that deconstruction can recover 50–95% of a building’s materials, depending on the project^7^. This reclaimed material can be sold, generating revenue and reducing demand for new materials and the associated energy costs^8^. Moreover, avoiding landfill disposal can minimise costs, including landfill taxes.

Deconstruction may generate economic advantages by reusing resources, creating work opportunities and lowering the cost of new construction^9^. The construction of structures such as the pavilion at Glyndebourne Opera in Sussex (built using oyster shells and corks), the Villa Welpelloo in the Netherlands (built using reclaimed textile machinery and cable reels)^10^, the Brighton waste house (built using reclaimed carpet tiles and chalky soil)^11^ and the 59-story Montparnasse tower in Paris, to mention but a few were built using recovered materials from older buildings, this perhaps generates income for the owners of the reclaimed materials. Besides, many of the used materials were collected using essential tools. It supposedly requires a larger workforce, often involving labour-intensive processes such as de-nailing, unscrewing, removing mortar from tiles, and cleaning marbles and slabs. As a result, potential jobs were created.

Deconstruction can also contribute to preserving built heritage, which carries significant social and economic benefits^12^. By carefully dismantling and repurposing materials, cities can retain historic structures that contribute to cultural identity and community pride. For example, Britain’s iconic castles and cathedrals symbolise the nation’s rich history and attract millions of visitors annually, generating substantial revenue through tourism, hospitality, and related industries^13–15^.

Despite its benefits, deconstruction faces practical challenges due to conventional building designs prioritising ease of demolition over reusability. Initiatives like the London Circular Economy Statement and pre-demolition audits aim to address this by promoting waste reduction, reuse, and recycling. Sustainability certification systems such as BREEAM also incentivise deconstruction over demolition. However, these programs often require specialised expertise and can be costly, limiting their widespread adoption.

To bridge these gaps, researchers have introduced the concept of "building deconstructability," which evaluates the feasibility of deconstructing buildings nearing the end of their lifecycle. This approach aligns with audits but provides insights into the building’s potential for material recovery. Developing a predictive model for deconstructability can guide stakeholders in making informed decisions, promoting sustainable practices, and reducing waste.

While existing deconstructability prediction models (DPMs) predominantly focus on technical^16,17^ or economic factors^18,19^, they often fail to provide a holistic evaluation of deconstructability. Recent studies emphasise the importance of incorporating broader perspectives—such as social, environmental, legal, and scheduling dimensions—for a more comprehensive assessment^20–25^. Furthermore, despite the proven advantages of machine learning (ML) techniques over traditional statistical models in various fields—including building energy prediction^26,27^, construction project management and delay analysis^28^, and air pollution forecasting^29–31^—ML has seen limited application in DPM research. Notable exceptions include studies^32,33^ that employ ML methods tailored to modern buildings designed for deconstruction or are compliant with Building Information Modelling (BIM), and this neglects older buildings that were not designed for deconstruction or lack BIM compliance.

To address these gaps, this research aims to develop a machine learning (ML)-based DPM that incorporates technical, economic, social, environmental, legal, and scheduling dimensions. This model will apply to BIM and non-BIM-compliant buildings and structures designed for deconstruction (DfD) and conventional buildings. The research seeks to answer the following questions: Which ML model demonstrates superior predictive generalizability for deconstructability prediction using data of diverse variables across technical, economic, social, environmental, legal, and scheduling dimensions? What are the key variables contributing to models with higher predictive generalizability?

For clarification, terms such as demolition, deconstruction, deconstructability and machine learning are defined as follows: *Demolition eliminates all building parts at a specific location and time*^34^. In another study by^9^, demolition was described as an engineered process to knock down buildings into debris.

*Deconstruction* is carefully knocking down a building into its components to rescue its materials for recycling, reuse, and reconstruction reasons^35^.

Deconstruction is a means to an end. It exists for the appropriate recovery of building elements, components, sub-components, and materials for reuse in the most cost-effective manner^36^.

*Deconstructability is the* feasibility/practicality of deconstructing buildings^16,23,37^. It incorporates material and component reusability and aims to determine whether deconstructing a building offers advantages over conventional demolition.

Machine learning (ML) is a type of Artificial intelligence where computers learn from sample data (train) to predict unseen data (test/validation)^24^. With the capability of finding unknown patterns in the data, ML can solve various problems, such as discovering associations between variables, making predictions based on criteria, recommending certain activities and identifying objects with similar patterns; readers interested in different ML^24^.

The remaining section includes variables influencing deconstructability and role of prediction models ("Variables influencing deconstructability and the role of prediction models" section), methodology ("Methodology" section), result and discussion ("Results" section) and conclusion and future research.

## Variables influencing deconstructability and the role of prediction models

### Existing deconstructability models

Models exist to predict deconstructability, but most are limited in scope. For example, Guy^37^ developed an evaluation tool focused on economic drivers, such as material quality and disposal costs, to estimate material reuse potential. Similarly, Akinade et al.^38^ proposed a design-based deconstructability assessment score (DAS), emphasising technical factors like material composition, joint types, and structural design. However, these models typically prioritise one dimension, such as technical or economic variables, and lack a broader, multidimensional perspective.

In recent years, machine learning (ML) techniques have gained traction for predictive modelling in deconstruction. For instance, Rakshan et al.^18^ employed 12 supervised ML methods, including parametric (e.g., Linear discriminant analysis, quadratic discriminant analysis, Naïve Bayes, and artificial neural network) and non-parametric (e.g., K-nearest neighbour, Decision tree, Random Forest, adaptive boosting, Support vector machine among others) to predict the reusability of structural components using economic variables such as labour and procurement costs. Another study by Rakshan et al.^39^ utilised the same 12 ML models, as in^18^, to analyse hazardous material content, emphasising technical factors. These models demonstrate the potential of ML to enhance predictive accuracy. Still, their limited focus on specific dimensions restricts their generalizability.

### Fundamentals of prediction models

Prediction models, particularly those based on ML, rely on analysing patterns within data to predict outcomes. These models can be supervised, unsupervised, or reinforcement learning, depending on the prior knowledge of the expected output for a given input^24^. Supervised learning is chosen for this study because it leverages labelled datasets where input features and corresponding outputs, such as deconstructability scores, are known. Supervised ML models consist of three primary components:

1. *Input Variables (Features)*: Variables influencing the prediction target (e.g., material composition, labour costs, connection type, among others). Selecting relevant features is crucial for the model’s performance^28–31,40^.
2. *Algorithm (Learning Method)*: The mathematical framework used to process data and identify patterns. Common ML algorithms include decision trees, random forests, support vector machines, and neural networks, each suited to different data types and objectives^18^.
3. *Output (Prediction Target)*: The variable the model aims to predict, such as a deconstructability score or material recovery rate.

ML-based prediction models offer several advantages, including handling large datasets, capturing complex relationships among variables, and generating insights that might be overlooked in traditional methods^24^.

### Justification for the proposed method

This research employs a machine learning-based Deconstructability Prediction Model (DPM) designed to address the limitations of existing approaches. Several considerations drive the choice of ML over traditional statistical models:

- *Multidimensionality*: ML can incorporate various technical, economic, social, environmental, legal, and scheduling dimensions to provide a holistic assessment^24^.
- *Complex Relationships*: ML algorithms can uncover nonlinear and interdependent relationships between features, often critical in deconstruction scenarios^22,24^.
- *Explainability*: By integrating feature importance analysis, the proposed ML model ensures transparency in understanding the relative impact of each variable on deconstructability predictions^28–30^.

A comprehensive understanding of deconstructability requires consideration of multiple dimensions influencing material recovery and reuse. Balogun et al.^6^ systematically reviewed 38 studies to identify six key dimensions: technical, economic, social, environmental, legal, and scheduling. This research follows the established classification framework, systematically categorising variables under these dimensions.

Social factors include public attitudes and local regulations, while economic variables assess financial feasibility, cost implications, and material reuse potential. Technical aspects focus on building design, modularity, and accessibility of structural components, whereas environmental considerations address sustainability practices, waste reduction, and ecological impacts. Scheduling factors concern project timelines, sequencing constraints, and logistical feasibility, while legal aspects cover regulatory compliance, building codes, and safety protocols.

To capture the complexity of deconstructability, the proposed ML-based DPM integrates these dimensions, incorporating technical (e.g., joint types, material reusability), economic (e.g., labour costs, landfill taxes), social (e.g., job creation, community engagement), environmental (e.g., carbon emissions), legal (e.g., compliance requirements), and scheduling (e.g., project duration) variables. By bridging these dimensions, the model provides a comprehensive tool for assessing deconstructability and addressing gaps in existing models. This approach enhances predictive accuracy and equips stakeholders with data-driven insights for informed decision-making in early deconstruction planning.

## Methodology

This paper presents the development of a machine learning-based deconstructability prediction model (ML-DPM) to assess building deconstructability. To achieve this, we utilised several ML models, drawing on methods from previous studies^18,39^. Given the lack of a dedicated database or repository for deconstruction projects, we employed an online survey methodology to gather data about past deconstruction projects from deconstruction professionals. The survey was based on established criteria and variables from a prior study^23^. This data was then used to both develop and validate the ML-DPM. Please refer to Appendix [A1] for details on the questionnaire and the established variables under different dimensions. To provide a detailed analysis of the relationships between these variables and their respective dimensions, a comprehensive table is presented in Appendix [A2]. This table explicitly maps each questionnaire variable to its relevant dimension(s), ensuring clarity in the classification and aiding in a holistic assessment of deconstructability. Integrating these dimensions is essential for developing predictive models optimising material recovery and reuse in deconstruction projects.

The survey link was disseminated electronically to respondents (deconstruction professionals) from November 2021 to June 2022. A wide range of professional bodies, groups, forums, and companies within and outside the United Kingdom (UK) were contacted to identify potential respondents. These included organisations such as the Institute of Demolition Engineers (IDE), Sustainability & Green Building, Build Reuse, Green Building Initiatives, Green Globes, Sustainable Construction, Rebuilding Warehouse, Home Builders Federation, Deconstruct UK, Skanska, and World Green Building Council, among others.

In total, 2831 prospective deconstruction professionals were contacted. After several back-and-forth reminders, 301 responded to the questions. 263 professionals were confirmed to have previously worked on deconstruction projects, representing the valid data points retrieved—indicating 263 deconstruction projects. The data recovered is sufficient and satisfactory for analysis, following the central limit theorem requirement of 30 samples^41,42^. In addition, the samples considered in this paper exceed those used in similar research^39,43^.

To investigate the questionnaire’s reliability, the responses’ internal consistency was assessed by computing Cronbach’s alpha value, as outlined in^28^. Before proceeding with model development using the collected data, the authors analysed the survey’s reliability using SPSS version 28. It’s worth noting that a Cronbach’s alpha value of 0.7 indicates acceptable reliability, while values higher than 0.7 up to 0.9 are considered more desirable^22^. The results of the reliability analysis revealed that Cronbach’s alpha value for this survey met the minimum requirement of 0.7, indicating satisfactory internal consistency.

**Fig. 1:**
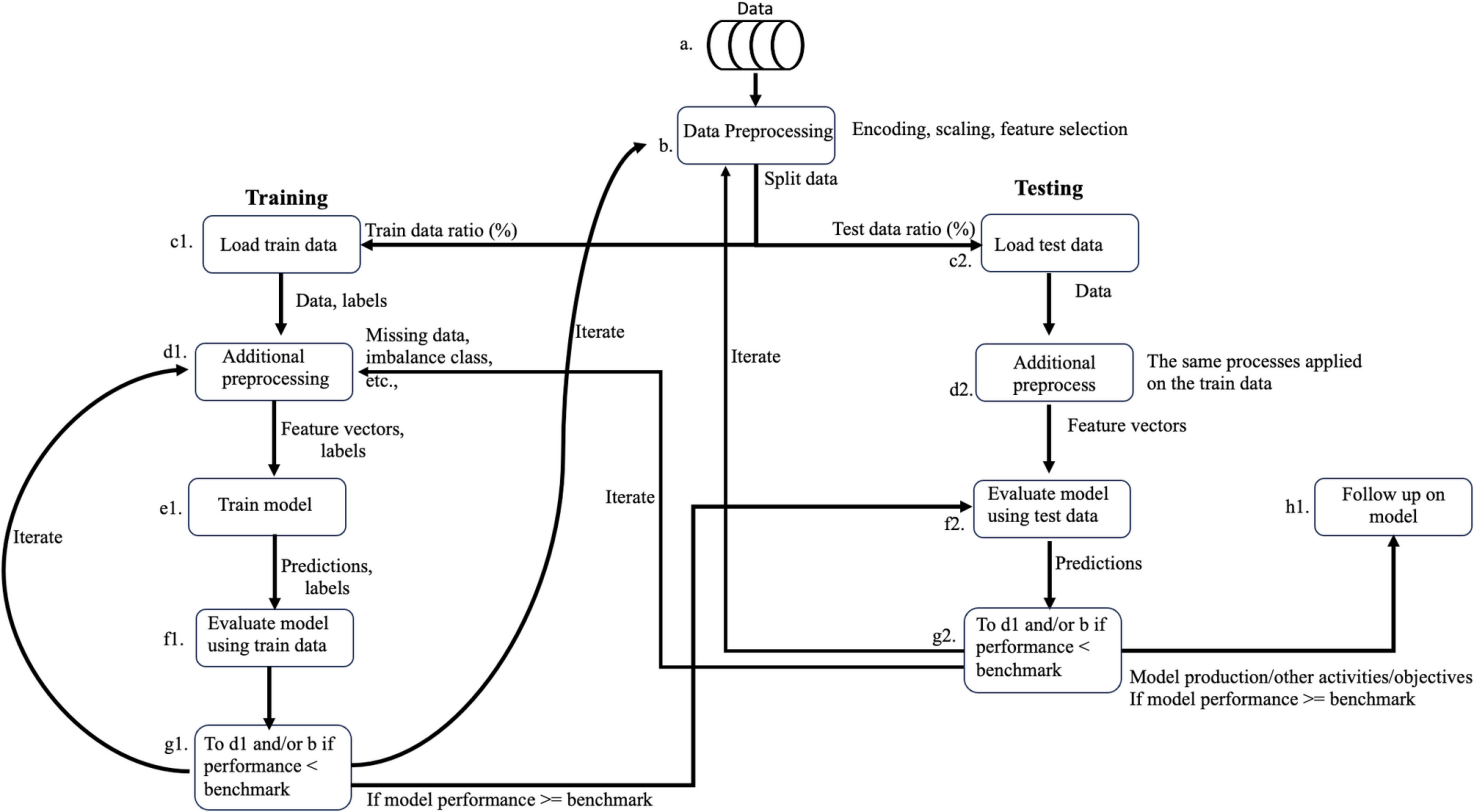
Supervised learning workflow.

Given the data’s characteristics and the discussion on deconstructability, this study falls under supervised learning, specifically binary classification, as deconstructability is categorised as either deconstructible or non-deconstructible. The workflow for supervised machine learning is divided into two main stages: training and testing. These stages can be iterative, allowing for continuous model refinement. Figure 1 shows a simple supervised learning workflow.

A training set is utilised to develop the classification model during the training phase. This involves selecting relevant features, choosing predictive models and/or tuning hyperparameters to achieve optimal performance. Once the model is trained, it is evaluated using a test set. The test set, which consists of data unseen by the trained model, is essential for assessing the model’s performance and generalizability. This evaluation helps identify potential overfitting and ensures the model performs well on unseen/new data. Adjustments can be made to improve the model’s accuracy and robustness by iterating between training and testing. While Fig. 1 presents a generic workflow, the step-by-step approach in this paper is explained as follows.

Data preprocessing: The data preprocess involves transforming questionnaire survey data into an artificial intelligence and machine learning predictive model-ready state. This preprocess involves handling missing value, scaling, discretisation, and feature engineering (i.e., feature selection, feature extraction/dimensionality reduction)^44^. Subsequent sections will discuss data encoding, missing data, and feature selection, amongst others, in detail.

Data encoding: ML models are mostly designed for numerical inputs; as a result, efficiently encoding categorical features is a significant step in data preprocessing. Readers interested in encoding techniques are referred to^45^. Among the various techniques available, one-hot encoding—a target-agnostic method—has been established as a standard approach for handling categorical variables^46,47^ and was used in this paper.

Missing Data: Data availability, quality, and completeness are common challenges in real-life ML model development. The same challenge was faced in this paper. Data for building deconstruction projects is not readily available, making the authors opt for data on past deconstruction projects from professionals, which was not free from missing values.

There are several ways to deal with missing data; they can, however, be classified into two: ignore the missing data^48^, which may not be the solution in this paper, and the second is to use a value to make up for the missingness (imputation), and that was utilised in this paper. Furthermore, from existing literature, it was discovered that many imputation techniques were supplied based on statistics and ML techniques^49,50^. However, different studies have shown the usefulness of ML-based imputation techniques. Of all these ML-based imputation methods, the KNN-based method stands out^50^ and was employed in this paper.

**Fig. 2:**
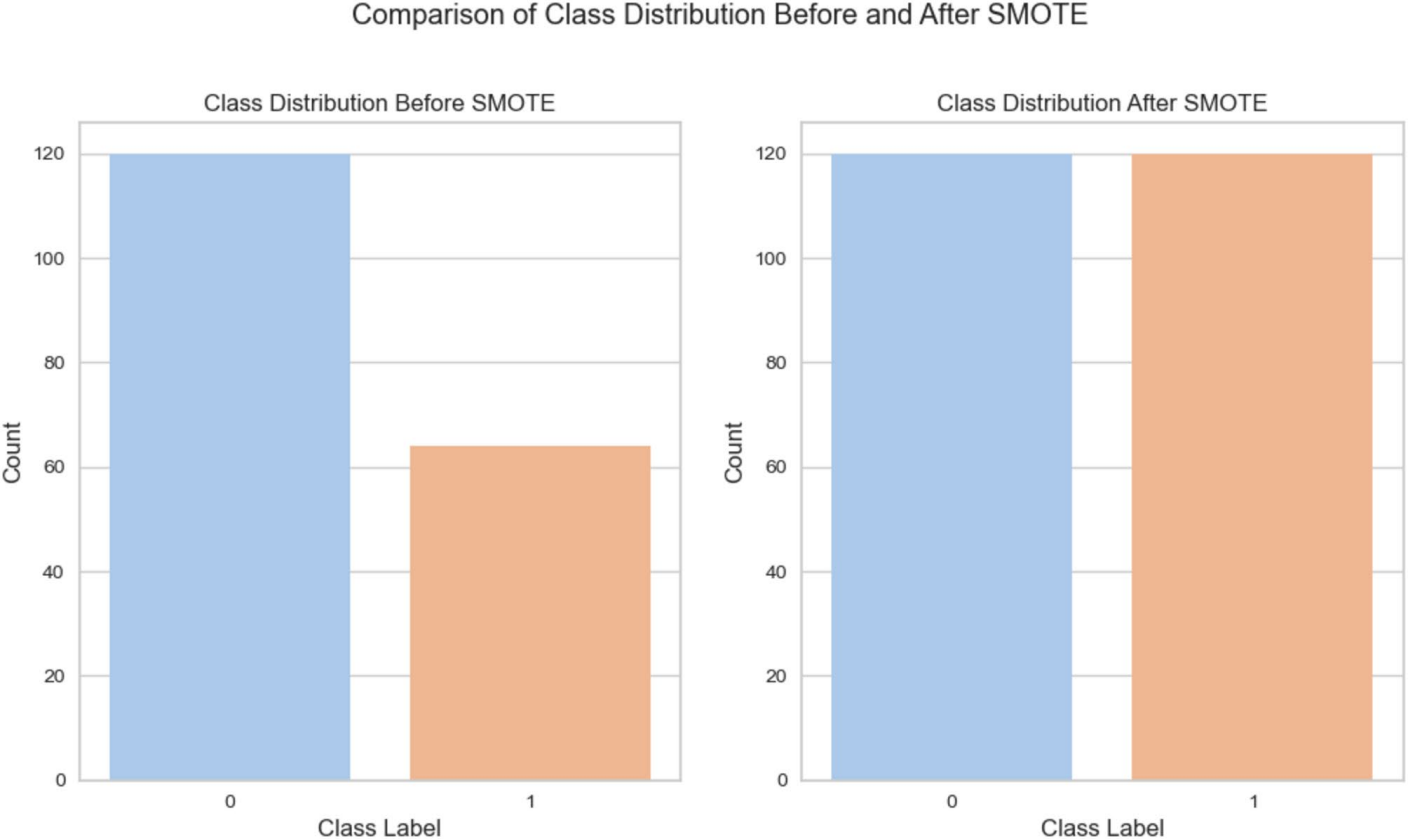
Class distribution before and after SMOTE (training set).

Imbalance Class: Unequal target class distribution is typical in real-life datasets, as seen in this paper, where the deconstructible (D) class is the minority. To address this, oversampling was employed, increasing the minority class samples. Specifically, the Synthetic Minority Over-sampling Technique (SMOTE) was employed. SMOTE identifies the nearest neighbours of minority samples using K-nearest neighbour (KNN)^51^. Following studies like^18,39^, this paper applied SMOTE due to its efficiency^52^. To avoid data leakage, a stratified train-test split was performed (75% training, 25% testing). SMOTE was then applied to the training data. See Fig. 2 for the class distribution before and after SMOTE.

Feature selection (FS): The supervised learning model’s performance depends on the input (i.e., features/variables). Research studies such as^28–31,40^ have proved the relevance of FS in different domains. It selects the most impactful features from the original set of features as new input features. Thus, not all features impact prediction, making FS critical in developing ML models. FS can help eliminate redundant information and improve generalisation^53^. FS can be a filter, wrapper or embedded method.

The filter method uses statistical characteristics of the dataset, providing feature ranking as output and selecting features regardless of model. The wrapper methods evaluate individual feature subsets using learning algorithms. Embedded methods select features during model development. All FS methods have their strengths and weaknesses and thus would have a different impact on the performance of the ML model. This paper employed an ensemble technique using the eight FS (three filters, three wrappers and two embedded methods).

*Filter methods*

1. Chi-square (CHI) is a filter method used to test independence. It evaluates the degree of association between variables by measuring deviation from the expected frequency^54^.
2. Analysis of Variance (AOV) is a univariate technique that utilises variance to detect the separability of each feature between classes^55^.
3. Mutual Information (MI) is used to evaluate the mutual dependence between variables^56^.

*Wrapper Methods*

4. Recursive feature elimination (RFE) recursively eliminates 0 − n features in each iteration, selecting an optimal number of features for each model^57^. Herein, a random forest classifier was used.
5. Forward Feature Selector (SFS) is an iterative technique that begins with no features. Initially, the feature with the best performance is added. Then, the next most significant feature that improves performance in combination with the previously added feature is selected. This process continues until a new feature no longer enhances the classifier’s performance^57,58^. Herein, a random forest classifier was employed.
6. Backward Feature selection (BFS) starts with all available features and recursively discards the most insignificant feature from the model. This elimination process is repeated until removing features does not enhance the model’s performance^58,59^. A logistic regression classifier was utilised as BFS.

*Embedded Methods*

7. Random Forest was used as an embedded feature selection method. The significance of each feature is calculated by performing random permutations of the features in the out-of-bag (OOB) set and measuring the increase in misclassification compared to the default state of the OOB set^60^.
8. LightGBM classifier (ELG) was utilised as an embedded feature selection method due to its efficiency^53^. ELG assigns global importance to features by averaging them across all trees (base learners).

Table 1 provides a comprehensive list of the features chosen to develop the ML-DPM through an ensemble technique for the FS. Features were selected based on their counts and included if their count satisfied at least 3/4 of the eight FS, meaning it was selected if a variable had a count of 6 or more (see Table 1).

**Table 1:** Feature selection methods, variables, and selection decision.

Variables	CHI	AOV	MI	RFE	FFS	BFS	ERF	ELG	Count	Decision
B.1			X	X			X	X	4	–
B.2		X			X	X		X	4	–
B.3					X				1	–
B.4	X		X	X				X	4	–
B.5		X			X	X		X	4	–
B.6	X		X	X	X		X	X	6	Selected
B.7	X	X	X	X			X	X	6	Selected
B.8			X	X	X		X	X	5	–
B.9	X	X	X	X		X	X	X	7	Selected
B.10								X	1	–
B.11					X	X			2	–
B.12								X	1	–
B.13					X	X			2	–
B.14		X				X		X	3	–
B.15				X		X	X		3	–
B.16				X	X		X		3	–
B.17									0	–
B.18		X			X	X		X	4	–
B.19	X	X	X	X		X	X	X	7	Selected
B.20		X				X		X	3	–
B.21						X		X	2	–
B.22					X	X			2	–
B.23					X	X			2	–
B.24		X			X	X		X	4	–
B.25	X	X	X	X	X	X	X	X	8	Selected
B.26					X	X			2	–
B.27	X	X		X		X	X	X	6	Selected
B.28	X	X	X	X	X	X	X	X	8	Selected
B.29					X				1	–
B.30									0	–
C.1			X	X	X		X	X	5	–
C.2	X	X	X	X			X	X	6	Selected
C.3			X	X			X	X	4	–
C.4	X		X	X			X	X	5	–
C.5	X	X	X	X	X		X	X	7	Selected
C.6	X	X	X	X			X	X	6	Selected
C.7	X	X	X	X			X	X	6	Selected
C.8	X	X	X	X	X	X	X	X	8	Selected
C.9					X			X	2	–
C.10				X	X	X	X		4	–
C.11					X				1	–
C.12				X	X		X		3	–
C.13			X	X	X		X		4	–
C.14	X	X	X	X	X	X	X	X	8	Selected
C.15	X	X	X	X		X	X		6	Selected
C.16						X			1	–
C.17			X	X			X	X	4	–
C.18			X	X		X	X	X	5	–
C.19	X		X	X	X		X	X	6	Selected
C.20	X	X	X	X	X	X	X	X	8	Selected
D.1	X	X			X			X	4	–
D.2	X	X	X					X	4	–
D.3									0	–
D.4	X	X	X		X				4	–
D.5	X	X			X				3	–
D.6	X	X							2	–
D.7	X	X	X						3	–
D.8									0	–
D.9	X	X	X						3	–
D.10	X	X			X				3	–
D.11	X	X	X	X		X	X	X	7	Selected
D.12	X	X	X	X		X	X		6	Selected
D.13	X	X			X				3	–
D.14	X	X	X			X			4	–
D.15									0	–
D.16	X	X							2	–
D.17	X	X	X			X			4	–
D.18					X	X			2	–
D.19	X	X	X			X			4	–
D.20	X	X							2	–
E.1			X	X		X	X		4	–
E.2	X	X	X	X			X		5	–
E.3			X	X			X		3	–
E.4	X		X	X	X		X		5	–
E.5	X	X	X	X	X		X		8	Selected
E.6	X	X			X	X			4	–
E.7	X	X	X			X			4	–
E.8				X		X	X		3	–
E.9	X	X	X			X			4	–
E.10	X	X			X	X			4	–
E.11						X			1	–
E.12					X	X			2	–
E.13					X				1	–
E.14									0	–
E.15						X			1	–
F.1				X		X	X	X	4	–
F.2	X	X	X	X	X		X	X	7	Selected
F.3	X	X	X		X	X		X	6	Selected
F.4		X				X		X	3	–
F.5					X				1	–
G.1				X	X	X	X	X	5	–
G.2	X		X	X			X	X	5	–
G.3			X	X	X	X	X	X	6	Selected
G.4				X			X	X	3	–
G.5	X		X	X			X	X	5	–
G.6				X	X		X		3	–
Total	45	45	45	45	45	45	45	45		22

22 variables were identified as having a count of 6 or more (Table 1), which is 3/4 of the total, i.e., 8. This threshold was chosen to balance the strengths and weaknesses of the eight feature selection methods. These selected variables will be used to develop ML-DPM. Furthermore, all variables will be tested for experimentation. This approach aims to achieve optimal model performance and identify the most impactful variables influencing deconstructability.

Models Training: The process of choosing a machine learning methodology for predictive modelling is paramount due to the absence of a universally superior model that suits all problems^26,27,40,61^. This paper aims to develop an ML model with the highest possible accuracy for estimating the deconstructability of a building. While accuracy is a pivotal criterion for model selection, the interpretability of the chosen model is equally crucial^18,39^. This holds weight in the context of this study, which strives to offer a comprehensible model accessible to diverse stakeholders in the deconstruction space. Notably, these stakeholders may lack the proficiency to navigate intricate predictive models. The insistence on interpretability is strategic, fostering effective utilisation of the model by stakeholders. This emphasis on accessibility is indispensable for the selected predictive model to fulfil its intended purpose.

After considering the preceding discussion, it seems reasonable to opt for interpretable models to train the predictive deconstructability model. However, it is essential to acknowledge that while interpretable models offer transparency, they may not consistently deliver high accuracy. Conversely, complex models, often called black boxes, provide accurate predictions. Nonetheless, it introduces challenges, including a loss of interpretability, increased variance, and the risk of overfitting, ultimately leading to a lack of generalisation of unseen data. Hence, when selecting a method for predictive modelling, careful consideration of the trade-off is crucial.

Moreover, as described in the existing literature, it’s a widely accepted practice to explore a wide range of ML models, such as Support Vector Machines and random forests. Although certain models may appear more suitable in theory or practical applications for specific tasks, our current understanding of these models does not reliably predict their performance in advance. The "no-free-lunch" theorems^62^ further emphasise that no single ML model consistently outperforms all others across diverse scenarios/domains. Therefore, when addressing complex problems like the one in this paper, it is advisable to experiment with multiple models to ascertain the most effective one.

Model Evaluation: ML models often do not perform as expected, necessitating evaluation. Predictions fall into four categories for binary classification, such as the classification of buildings as deconstructible (1) or non-deconstructible (0). Table 2 shows the confusion matrix, which includes false negatives (FN), where a deconstructible building is incorrectly labelled as non-deconstructible; false positives (FP), where a non-deconstructible building is labelled as deconstructible; true positives (TP), where a deconstructible building is correctly labelled; and true negatives (TN), where a non-deconstructible building is correctly labelled.

**Table 2:** Confusion matrix.

		Prediction	
		Negative (Not deconstructible)	Positive (Deconstructible)
Actual	Negative (Not deconstructible)	True Negative	False Positive
	Positive (Deconstructible)	False Negative	True positive

From Table 2, various metrics can be calculated, including accuracy, precision, recall, F1 score, ROC curve, and AUROC (Area Under the Receiver Operating Characteristic Curve). Higher scores for these metrics (closer to 1.0 or 100%) indicate a better predictive model. Readers interested in these metrics can check^31,40^. Their definitions are provided below.

$${\text{Accuracy }} = \frac{{{\text{True }}\;{\text{Negatives}} + {\text{True }}\;{\text{Positives}}}}{{{\text{Number}}\;{\text{ of}}\;{\text{ predictions}}}}$$

$${\text{Precision }} = { }\frac{{{\text{True}}\;{\text{ Positives}}}}{{{\text{True}}\;{\text{ positives }} + {\text{ False }}\;{\text{positives}}}}$$

$${\text{Recall }} = { }\frac{{{\text{True }}\;{\text{Positives}}}}{{{\text{True}}\;{\text{ positives }} + {\text{ False }}\;{\text{Negatives}}}}$$

$${\text{F}}1 = { }2{ * }\frac{{\text{Precision * Recall}}}{{{\text{Precision }} + {\text{ Recall}}}}$$

The Receiver Operating Characteristic (ROC) curve is a graphical plot illustrating the trade-off between the true positive rate (TPR) and the false positive rate (FPR). AUROC summarises the ROC curve.

Furthermore, the resampling technique is commonly used to evaluate ML and estimate how well it will generalise on test data. K-fold cross-validation is among the common and efficient techniques and was employed in this paper. In K-fold cross-validation (KfCV), the original dataset is randomly divided into K folds, each containing roughly equal observations. One of the K folds is set aside as the testing set, while the remaining k-1 folds are used for training the model. The model’s performance is then assessed using the held-out set, repeating this process k times, with each fold serving as the validation set. Performance metrics are recorded for each fold, and the model’s overall performance is evaluated by averaging across the k folds. Typical values are k = 5 and k = 10; we have used k = 5 in this paper.

Experiment: Two groups of datasets were used to develop the predictive model, from which the most efficient (generalisable) and explainable model will be chosen as the final ML-DPM. Group 1 uses all 96 variables; Group 2 uses 22 variables obtained through Ensemble FS.

We experimented to evaluate the performance of various machine learning models using a dataset split into training and testing sets. While different splitting ratios (e.g., 50:50, 60:40, 70:30, 80:20) are commonly used, no single ratio is universally best^63,64^. For this paper, we partitioned the dataset into 75% for training and 25% for testing, following studies such as^65,66^.

**Fig. 3:**
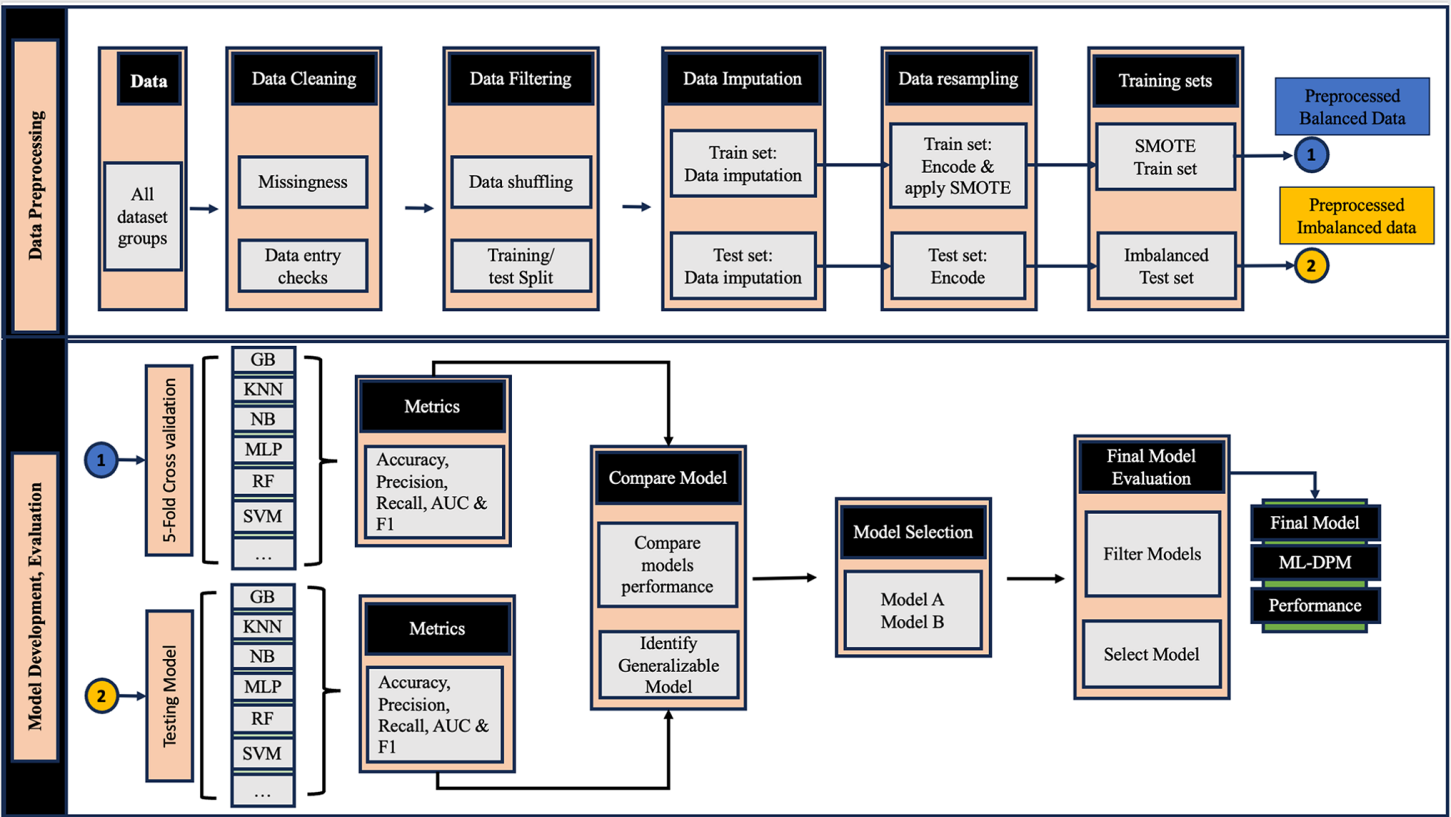
Workflow for ML-deconstructability predictive model.

We employed fivefold cross-validation (CV) to assess each model’s performance rigorously. During each CV iteration, the models were trained on the balanced training subset and tested on the imbalanced test set to provide robust and unbiased performance estimates. This approach ensured the oversampling process did not artificially inflate the models’ performance metrics. By testing on the untouched 25%, we assessed how well the models could generalise to real-world data distributions, thereby providing a realistic measure of their effectiveness. Figure 3 presents the overview of the ML-DPM development/validation workflow.

## Results

Table 3 illustrates how various classification algorithms (twelve of them selected as common and efficient^26–29,40^) performed across two distinct sets of features: utilising all features and features selected through FS. The confusion matrices, fivefold cross-validation (CV) on a 75% balanced training set, then tested on 25% of untouched data, were presented in Table 3. Predicted values (P) represented rows in each confusion matrix, and the columns indicated actual values (A). The categories within matrices were labelled "D" (Deconstructible) and “ND” (Non-Deconstructible), representing two possible classes.

**Table 3:** Confusion Matrix for the developed ML Models for balanced (SMOTE) and Imbalance data (Validation).

Model	All Features						Features from Ensemble FS					
	5-Fold CV (Balance data)			Testing (Imbalance data)			5-Fold CV (Balance data)			Validation (Imbalance data)		
Gradient boosting (GB)	P/A	ND	D	P/A	ND	D	P/A	ND	D	P/A	ND	D
	ND	97	35	ND	16	17	ND	87	45	ND	22	11
	D	47	85	D	7	26	D	45	87	D	6	27
K Nearest neighbour (KNN)	P/A	ND	D	P/A	ND	D	P/A	ND	D	P/A	ND	D
	ND	36	96	ND	11	22	ND	39	93	ND	4	29
	D	10	122	D	3	30	D	9	123	D	0	33
Naive Bayes (NB)	P/A	ND	D	P/A	ND	D	P/A	ND	D	P/A	ND	D
	ND	92	40	ND	26	7	ND	90	42	ND	23	10
	D	38	94	D	12	21	D	42	90	D	4	29
Artificial Neural Network (MLP)	P/A	ND	D	P/A	ND	D	P/A	ND	D	P/A	ND	D
	ND	95	37	ND	27	6	ND	98	34	ND	23	10
	D	43	89	D	7	26	D	40	92	D	7	26
Random Forest (RF)	P/A	ND	D	P/A	ND	D	P/A	ND	D	P/A	ND	D
	ND	101	31	ND	26	7	ND	108	24	ND	19	14
	D	43	89	D	9	24	D	45	87	D	8	25
Support vector Machine with Linear kernel (SVM-L)	P/A	ND	D	P/A	ND	D	P/A	ND	D	P/A	ND	D
	ND	74	58	ND	22	11	ND	90	42	ND	19	14
	D	50	82	D	8	25	D	44	88	D	8	25
Support vector Machine with polynomial kernel (SVM-P)	P/A	ND	D	P/A	ND	D	P/A	ND	D	P/A	ND	D
	ND	95	37	ND	23	10	ND	96	36	ND	12	21
	D	38	94	D	5	28	D	25	107	D	3	30
Support vector Machine with Radial kernel (SVM-R)	P/A	ND	D	P/A	ND	D	P/A	ND	D	P/A	ND	D
	ND	111	21	ND	31	2	ND	112	20	ND	29	4
	D	52	80	D	11	22	D	51	81	D	12	21
Logistic regression (LR)	P/A	ND	D	P/A	ND	D	P/A	ND	D	P/A	ND	D
	ND	82	50	ND	21	12	ND	96	36	ND	21	12
	D	47	85	D	8	25	D	42	90	D	8	25
AdaBoost (AB)	P/A	ND	D	P/A	ND	D	P/A	ND	D	P/A	ND	D
	ND	86	46	ND	21	12	ND	93	39	ND	18	15
	D	45	87	D	12	21	D	44	88	D	6	27
Discriminant Analysis (DA)	P/A	ND	D	P/A	ND	D	P/A	ND	D	P/A	ND	D
	ND	60	72	ND	27	6	ND	96	36	ND	15	18
	D	54	78	D	16	17	D	42	90	D	9	24
Decision Tree (DT)	P/A	ND	D	P/A	ND	D	P/A	ND	D	P/A	ND	D
	ND	80	52	ND	21	12	ND	86	46	ND	16	17
	D	49	83	D	9	24	D	52	80	D	12	21

Analysing the confusion matrices provides valuable insights into the model’s performance metrics, such as accuracy, precision, recall, and F1 score for each feature set. For example, in the first confusion matrix utilising all features, there were fewer correctly predicted instances (true positives and true negatives), with counts of 26 and 16, respectively. In contrast, the matrices for the reduced feature sets (features from Ensemble FS) showed higher counts of correctly predicted instances (i.e., 22 and 27 for Ensemble FS). This indicates that despite using fewer features in the ensemble FS sets, the models maintain high-performance levels, suggesting the efficiency of FS.

Overall, Table 3 provides a comprehensive comparison, illustrating that while all features might offer the best raw accuracy, carefully selected feature subsets, through Ensemble FS techniques, can achieve comparable performance with potentially improved interpretability and reduced computational cost.

**Fig. 4:**
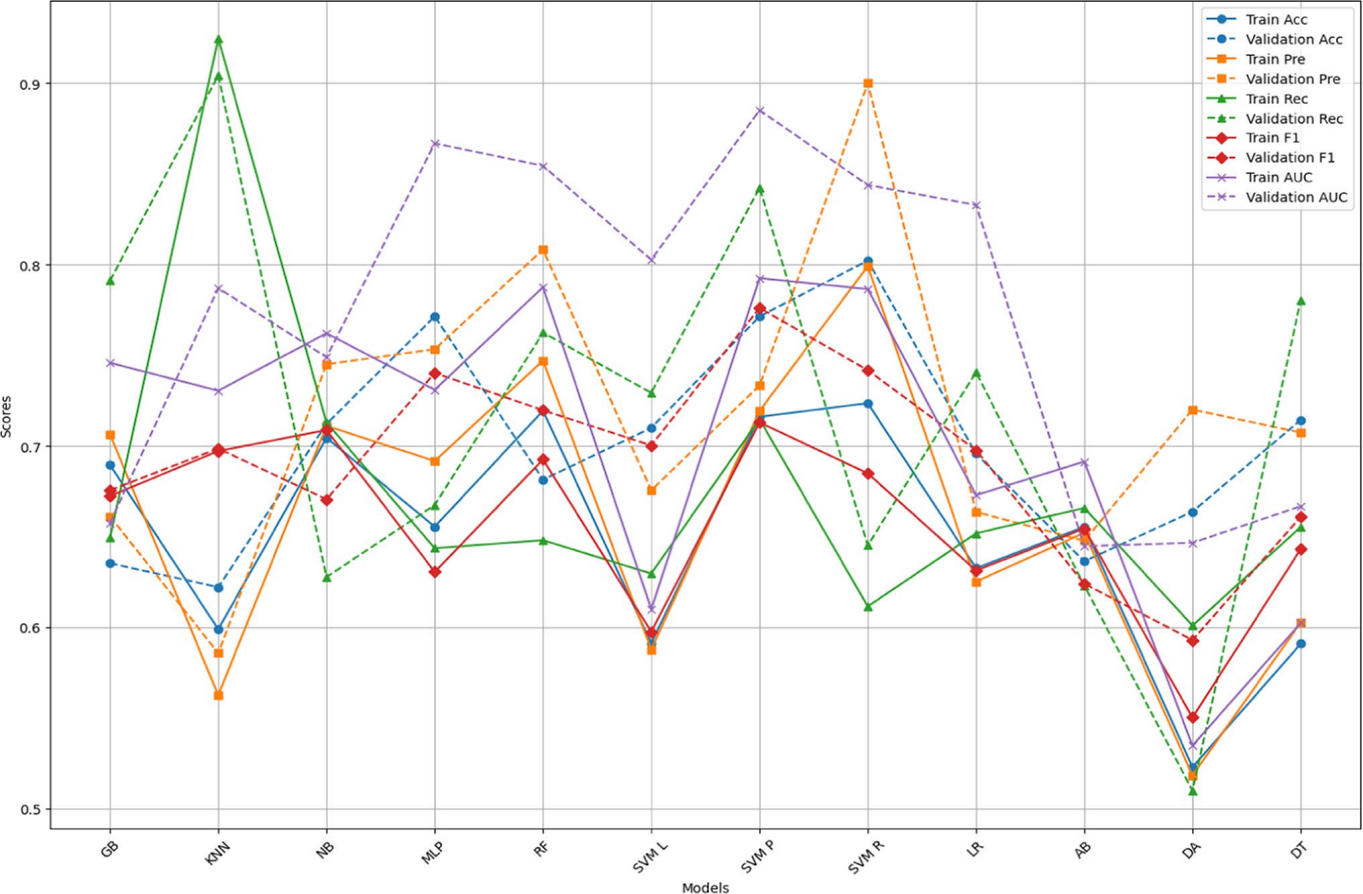
ML performance using all features.

**Fig. 5:**
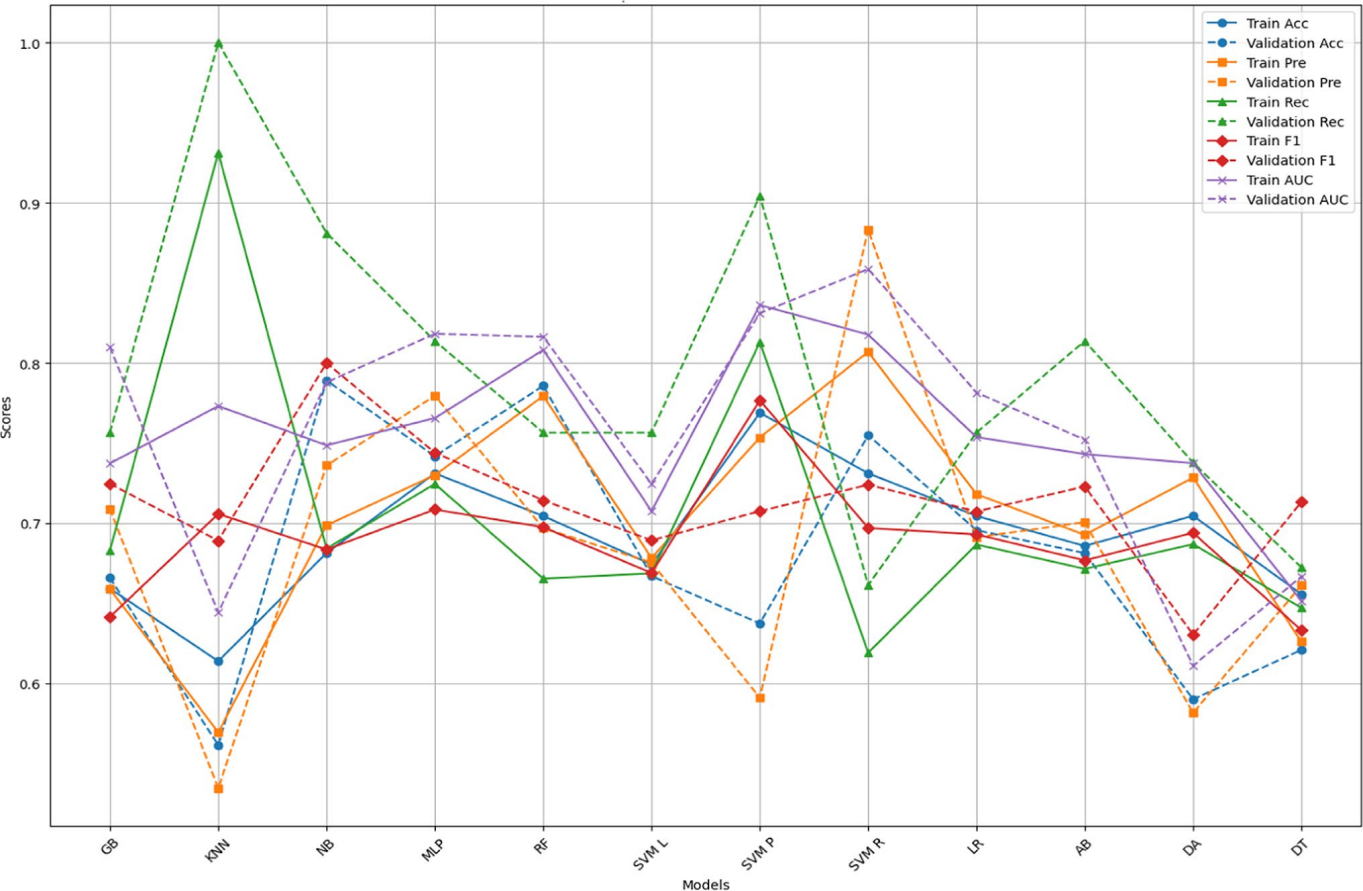
ML model performance using the features from the ensemble FS.

Figures 4 and 5 offer a comprehensive comparison of model performance across various evaluation metrics—Accuracy (Acc), Precision (Pre), F1 score (F1), Recall (Rec), and Area Under the Curve (AUC)—for different classification algorithms. These metrics were calculated through fivefold cross-validation and then tested on the untouched, imbalanced data (25% of the total dataset). Figures 4 and 5 correspond to the metrics calculated for the different feature sets: all features and features selected through the Ensemble FS technique, respectively.

Each Fig compares the metrics obtained from the CV phase with those from the testing phase. This comparison helps assess whether the model’s performance on the training data indicates its generalizability to unseen data. If the performance metrics from cross-validation exceed those from testing, it suggests potential overfitting, where the model learns to memorise the training data instead of learning underlying patterns. Conversely, suppose the performance metrics from testing are comparable or better than those from cross-validation. In that case, the model is not overfitting and can generalise well to new, unseen data. For example, Fig. 4 highlights instances of potential overfitting, where the model’s performance during cross-validation surpasses that of testing. In Fig. 4, the random forest achieved an accuracy of 0.7194 during cross-validation but decreased to 0.6813 during testing, indicating potential overfitting. Conversely, models like Gaussian Naive Bayes (NB), Logistic regression (LR) and Artificial neural network (MLP) demonstrate consistent performance across both phases, suggesting robust generalisation capabilities.

Based on performance metrics in Figs. 4 and 5, the best machine learning models can be identified by examining AUC, accuracy, precision, recall, and F1 scores. Importantly, we aim to select models that do not exhibit signs of overfitting.

From Fig. 4, the Support Vector Machine with Polynomial Kernel (SVM P) stands out with consistently high-performance metrics across cross-validation and testing phases. It achieves an AUC of 0.7925 and 0.8852, an accuracy of 0.716 and 0.7714, and a balanced precision, recall, and F1 score, indicating a robust and generalisable model. Artificial Intelligence with multi (MLP) also performs well with an AUC of 0.7309 and 0.8669, accuracy of 0.6554 and 0.7714 on cross-validation and testing, and balanced precision, recall, and F1 scores, showcasing strong generalisation. Lastly, the Support Vector Machine with radial kernel (SVM R) model is notable for its stable performance with an AUC of 0.7865 and 0.8439, accuracy of 0.7235 and 0.8022, and other metrics suggesting reliable performance on both balanced and imbalanced datasets.

From Fig. 5, MLP continues to excel with an AUC of 0.7656 and 0.8182, accuracy of 0.7311 and 0.7418, and high precision, recall, and F1 scores. This consistent performance across various feature sets makes it a top contender. The naive Bayes (NB) model also performs well with an AUC of 0.7486 and 0.7879, an accuracy of 0.6814 and 0.789, and balanced metrics, indicating it handles different feature sets effectively. Lastly, Gradient Boosting (GB) maintains strong performance with an AUC of 0.7373 and 0.8099, accuracy of 0.6592 and 0.6659, and other balanced metrics, making it a reliable choice.

Based on their consistent and robust performance across all metrics, the top two models were SVM with Polynomial Kernel (SVM P) for all features and Artificial intelligence with multilayer perceptron (MLP) using Ensemble FS. These models demonstrated generalisation capabilities without overfitting, making them suitable candidates for deployment. Table 4 presents the metrics (generated from the test set) of the top two models and the features used.

**Table 4:** Top two model’s performance.

Model	Features used	Accuracy	Precision	Recall	F1	AUC
SVM P	All features	0.7714	0.7333	0.8425	0.7761	0.8852
MLP	Features from Ensemble FS	0.7418	0.7795	0.8137	0.744	0.8182

From the two best, the SVM with a polynomial kernel stands out as a robust choice due to its well-rounded performance across multiple evaluation criteria. With an AUC of 88%, the model demonstrates excellent capability in distinguishing between the deconstructability classes, indicating a high overall effectiveness in prediction. Additionally, the SVM’s F1 score of 77% reflects a good balance between precision and recall, which is crucial for scenarios where false positives and negatives carry significant consequences. The recall rate of 84% suggests that the model is particularly adept at identifying the true positive cases (deconstructability class), reducing the risk of missing critical instances. However, the precision of 73% indicates there is some trade-off, with a moderate rate of false positives. Nevertheless, an accuracy of 77% shows that the model performs reliably across the entire dataset, making it a dependable choice for general use.

### Discussion

The findings in Table 3 provide a comprehensive evaluation of twelve machine learning models, comparing their performance across two feature sets: all features and those selected through Ensemble Feature Selection (FS). The analysis was conducted using fivefold cross-validation on a balanced training dataset, followed by testing on a 25% subset of untouched, more imbalanced data. The confusion matrices offer valuable insights into model performance, particularly regarding accuracy and generalizability for deconstructability predictions.

A noticeable trend emerged when comparing the models’ performance using all features versus those with features selected through Ensemble FS. While using all features generally led to the highest raw accuracy in most models, the reduced feature sets from Ensemble FS often resulted in similar, if not better, performance in correctly predicted instances. For example, MLP using Ensemble FS achieved higher correct predictions in both “Deconstructible” (D) and "Non-Deconstructible" (ND) categories compared to the model using all features. This suggests that even with a smaller set of features, feature selection can significantly enhance model efficiency without sacrificing predictive power. This is particularly advantageous in practical applications where computational efficiency is critical for decision-making.

Another key aspect of the evaluation was understanding the impact of overfitting. By comparing performance metrics from both cross-validation and testing phases—such as accuracy, precision, recall, F1 score, and Area Under the Curve (AUC)—overfitting was observed in specific models, particularly Random Forest (RF) and Support Vector Machines with Linear kernel (SVM-L). In these models, performance on the training dataset was significantly higher than on the test data, suggesting that they may have overlearned the training data and struggled to generalise to unseen data. In contrast, Support Vector Machines with Polynomial kernel (SVM-P) and Artificial Neural Networks (MLP) showed more consistent performance across both datasets, suggesting better generalisation capabilities.

This observation underscores the importance of model generalizability, which is crucial for real-world applications like deconstructability prediction. The consistent performance of SVM-P and MLP across training and testing datasets positions them as strong candidates for practical use, where robust and generalisable predictions are essential. Of the two models with the best generalisation and no overfitting, SVM-P was selected as the best due to its superior performance metrics and explainability, which are crucial for decision-makers and potential end users.

An important consideration in this context is misclassification. False classifications—predicting a building as deconstructible when it is not, or vice versa—could lead to unnecessary audits or missed opportunities for deconstruction. These misclassifications highlight the need for risk management strategies. For example, incorporating sustainability metrics like Building Research Establishment Environmental Assessment Method (BREEAM) ratings into deconstructability predictions could help mitigate potential costs and encourage more sustainable construction practices.

In practical terms, the models developed in this study—particularly SVM-P—could be integrated into tools for construction and real estate professionals. These tools could be an initial step in assessing a building’s deconstructability. By providing quick, cost-effective predictions, they can guide more detailed audits and help stakeholders, such as building owners and developers, make more informed decisions regarding the end-of-life treatment of structures. This process could help reduce demolition waste and promote resource reuse, furthering sustainability goals.

A potential real-world application could involve a building owner determining whether a structure can be deconstructed or must be demolished. The system could predict whether the building is deconstructible or non-deconstructible by inputting available building data into the ML model. If deconstructible, the owner would proceed with detailed disassembly planning. If non-deconstructible, the owner would proceed with demolition. This rapid assessment could save time and resources, contributing significantly to sustainability efforts in the construction industry.

### Conclusion

This study developed a machine learning-based deconstructability prediction model (ML-DPM) incorporating six key dimensions: technical, economic, social, environmental, legal, and scheduling. The primary goal was to identify the most significant explanatory variables influencing deconstructability while ensuring the model’s generalizability across diverse building types. The Support Vector Machine with a polynomial kernel (SVM-P) emerged as the most accurate and generalisable predictive model through extensive testing of various ML models. However, the research found that the 96 variables used in the model development were preselected based on expert input rather than derived from the machine learning process itself. This highlights that the study primarily evaluated the effectiveness of different ML techniques within a predefined variable set rather than discovering new explanatory variables.

The validation of these 96 variables in the context of the model’s performance underlines the importance of a holistic, multidimensional approach in predicting deconstructability. The results confirm that integrating multiple dimensions—spanning technical, economic, social, environmental, legal, and scheduling factors—substantially enhances predictive accuracy. However, the study also points out that future research should explore alternative variable selection strategies, including data-driven feature discovery, to potentially uncover additional or refined variables that may further enhance the model’s explanatory power.

In practical terms, this ML-DPM can serve as an early-stage decision-support tool for stakeholders involved in deconstruction projects, facilitating more informed and efficient assessments and optimising resource reuse. While the model demonstrates strong predictive performance, further refinement and real-world validation using additional datasets are recommended to improve its applicability across different building types and geographic contexts.

### Limitation and future research

The use of a questionnaire was a key limitation in this paper. First, the data was sourced from deconstruction professionals; the self-reported nature of surveys introduces potential biases. Respondents’ subjective experiences, particularly regarding intangible factors like economic viability and environmental impact, may have compromised the accuracy of the data. Second, the survey may not fully represent global building types or construction practices. Responses were likely concentrated in specific regions, limiting the model’s applicability across diverse cultural, regulatory, and technological contexts. Certain building types, especially in less-developed regions, may also be underrepresented, impacting the model’s prediction accuracy for these categories. Overall, the survey data laid a strong foundation for the ML-DPM; its limitations highlight the need for a more diverse, precise, and dynamic dataset in future studies.

Furthermore, oversampling techniques to augment the imbalance class was another limitation, even though we have tried to reduce this by applying SMOTE only to the training set to avoid data leakage. Future research should investigate the possibility of getting more data, including video, images, and documents, to develop ML-DPM. They should also seek to carry out large-scale ML-DPM validation through expert evaluation/practical implementations. Collaborating with industry professionals and using a hands-on approach can uncover nuances and context-specific factors that might not be evident through theoretical modelling.

## Electronic supplementary material

Below is the link to the electronic supplementary material.

Supplementary Material 1

Supplementary Material 2

## Data Availability

Data is provided within the supplementary information files.
