# Supplementary material for: Deconstructability prediction for building using machine learning and ensemble feature selection techniques
Source: Sci Rep. 2025 Jul 1;15:22152. doi: 10.1038/s41598-025-00790-0 (PMC12215758; doi:10.1038/s41598-025-00790-0)
Supplement: Supplementary file 2 — Supplementary Material 2 [file 41598_2025_790_MOESM2_ESM.docx]

## Appendix A: Online Survey

*Table A1. List of Online survey questions*

| **Question Number** | **Question** | **Question type** | **Options** |
| --- | --- | --- | --- |
| **Section A** | | | |
| A1.1 | ﻿I confirm that I have read and understood the  respondent information sheet | ﻿Multiple Choice: Single Answer | ﻿Yes / No |
| A1.2 | ﻿I understand that all personal information will remain confidential and that all efforts will be made to ensure I cannot be identified (except as might be required by law). | Multiple Choice: Single Answer | Yes / No |
| A1.3 | ﻿I agree that data gathered in this study may be stored securely and anonymously and may be used for solely for this research | Multiple Choice: Single Answer | Yes / No |
| A1.4 | ﻿I understand that my participation is voluntary and that I am free to withdraw at any time without giving a reason. | Multiple Choice: Single Answer | Yes / No |
| A1.5 | ﻿I agree to take part in this study | Multiple Choice: Single Answer | Yes / No |
| A1.6 | I have worked/participated/engaged on/in a building end-of-life project where disassembly/deconstruction was considered/done? | Multiple Choice: Single Answer | Yes / No |
| **NOTE**: If your response to A1.6 is no, then you are not fit to respond to the subsequent questions. Thank you and Goodbye. | | | |
| A1.7 | If response to A1.6 is yes, what option was agreed upon/implemented | Multiple Choice: Single Answer | Deconstruction / Refurbishment /  Renovation / Demolition / Abandon or Do nothing |
| Respondents Information | | | |
| A1.8 | ﻿What of the following services do you offer? | ﻿Multiple Choice: Multiple  Answers | Demolition / Deconstruction / Refurbishment / Redevelopment / Others |
| A1.9 | ﻿How would you best describe your profession/role? | Multiple Choice: Single Answer | Client / Developer / Demolition Contractor or Engineer / Architect / Other |
| A1.10 | How many years of experience do you have in Demolition/Deconstruction/waste management field? | Multiple Choice: Single Answer | 1 / 2 / 3 / 4 / 5+ |
| **NOTE:** Where the respondent has worked on multiple deconstruction/demolition project, s/he should please provide answers based on just one project. However, respondents can complete this questionnaire multiple times; in each case, all answers should be based on a specific project. | | | |
| A1.11 | I confirm that all/subsequent response is exclusively focused on a single deconstruction project? | Multiple Choice: Single Answer | Yes / No |
| A1.12 | To what extent was the building deconstructed or can the building be deconstructed to? | Multiple Choice: Single Answer | Full deconstruction / Partial demolition / Full Demolition |
| A.1.13 | On a scale of 0 -100, can you describe how deconstructible the building was? | Multiple Choice: Single Answer | < 25% / 25-50% / 50-99% / 100% |
| **Section B** | | | |
| B.1 | Where was/is the building deconstruction project located? | Multiple Choice: Single Answer | USA / UK / France / China / Others |
| B.2 | What was/is the building type? | Multiple Choice: Single Answer | Residential / Non-residential |
| B.3 | What was/is the construction method of the building? | Multiple Choice: Single Answer | Prefabricated / Traditional |
| B.4 | What year was/is the building built? | Multiple Choice: Single Answer | pre-1990 / pre-1930 / pre-1950 / pre-1978 / post-1978 |
| B.5 | Was/is the building Occupied | Multiple Choice: Single Answer | Yes / No |
| B.6 | Number of stories/floors | Multiple Choice: Single Answer | 1 / 1½ / 2 / 3 / More |
| B.7 | Numbers of rooms | Multiple Choice: Single Answer | 1 / 2 / 3 / 4 / 5+ |
| B.8 | Number of bathrooms/toilets | Multiple Choice: Single Answer | 1 / 2 / 3 / 4 / Others |
| B.9 | Was/is the structure secured to prevent unwanted entry? | Multiple Choice: Single Answer | Fully / Partly / No |
| B.10 | Was/is there room around the structure to serve as staging area? | Multiple Choice: Single Answer | Yes / No |
| B.11 | Was/is there exterior trash? | Multiple Choice: Single Answer | No trash / Piles of Trash |
| B.12 | Was/is there interior trash? | Multiple Choice: Single Answer | No trash / Piles of Trash |
| B.13 | Were there restricted movement in and out of the building? | Multiple Choice: Single Answer | Yes / No |
| B.14 | Were hazards present on-site? | Multiple Choice: Single Answer | Yes / No |
| B.15 | What was/is the state of the building? | Multiple Choice: Single Answer | Collapse / Partial collapse / Healthy |
| B.16 | Was/is the structural elements connection accessible/separable? | Multiple Choice: Single Answer | Yes / No |
| B.17 | Was/is there good road network to the building? | Multiple Choice: Single Answer | Yes / No |
| B.18 | What was/is the roof type? | Multiple Choice: Single Answer | Flat / Pitched |
| B.19 | What was/is the foundation type? | Multiple Choice: Single Answer | Monolithic concrete / Concrete block / Combination / Unknown / Others |
| B.20 | Was/is there any recycling facility closer? | Multiple Choice: Single Answer | Yes / No |
| B.21 | Was/is there major cracking of brick/wood rotting? | Multiple Choice: Single Answer | Yes / No |
| B.22 | Was/is there broken or missing windows? | Multiple Choice: Single Answer | Yes / No |
| B.23 | Was/is there missing bricks and siding | Multiple Choice: Single Answer | Yes / No |
| B.24 | Was/is there roof damage | Multiple Choice: Single Answer | Yes / No |
| B.25 | Were there major fire damages? | Multiple Choice: Single Answer | Yes / No |
| B.26 | Were there major water damages? | Multiple Choice: Single Answer | Yes / No |
| B.27 | What hazardous materials were identified? | Multiple Choice: Single Answer | Asbestos / Mercury / Lead / Others |
| B.28 | What is the estimated quantity of hazardous materials identified? | Multiple Choice: Single Answer | 1 (little) / 2 / 3 / 4 / 5 (lots) |
| B.29 | Do you have access to information about the building? (design plans and/or inventory) | Multiple Choice: Single Answer | Yes / No |
| B.30 | Has a detailed disassembly plan been developed? | Multiple Choice: Single Answer | Yes / No |
| **Section C** | | | |
| C.1 | What was/is the percent of Brick Siding? | Multiple Choice: Single Answer | 1 (little) / 2 / 3 / 4 / 5 (lots) |
| C.2 | What was/is the percent of Wood Siding? | Multiple Choice: Single Answer | 1 (little) / 2 / 3 / 4 / 5 (lots) |
| C.3 | What was/is the percent of Stone Siding? | Multiple Choice: Single Answer | 1 (little) / 2 / 3 / 4 / 5 (lots) |
| C.4 | What was/is the percent of Vinyl/Synthetic Siding? | Multiple Choice: Single Answer | 1 (little) / 2 / 3 / 4 / 5 (lots) |
| C.5 | What was/is the percent of Aluminium Siding? | Multiple Choice: Single Answer | 1 (little) / 2 / 3 / 4 / 5 (lots) |
| C.6 | What was/is the percent of other siding? | Multiple Choice: Single Answer | 1 (little) / 2 / 3 / 4 / 5 (lots) |
| C.7 | What was/is the number of rooms with wood flooring? | Multiple Choice: Single Answer | 1 / 2 / 3 / 4 / 5+ |
| C.8 | Was/is there dimensional ceiling or floor joists observed? | Multiple Choice: Single Answer | Yes / No / Unknown |
| C.9 | Was/is there dimensional lumber larger than 4x4? | Multiple Choice: Single Answer | Yes / No / Unknown |
| C.10 | What was/is the percent of Wall plasters? | Multiple Choice: Single Answer | < 25% / 25-50% / 50-99% / 100% |
| C.11 | What was/is the percent of drywall? | Multiple Choice: Single Answer | < 25% / 25-50% / 50-99% / 100% |
| C.12 | What was/is the number of rooms with crown moulding? | Multiple Choice: Single Answer | 1 / 2 / 3 / 4 / 5+ |
| C.13 | What was/is the number of rooms with casing around doors & windows? | Multiple Choice: Single Answer | 1 / 2 / 3 / 4 / 5+ |
| C.14 | What was/is the number of rooms with baseboard moulding? | Multiple Choice: Single Answer | 1 / 2 / 3 / 4 / 5+ |
| C.15 | What was/is the number of rooms with chair railing moulding? | Multiple Choice: Single Answer | 1 / 2 / 3 / 4 / 5+ |
| C.16 | Was/is there basement? | Multiple Choice: Single Answer | Yes / No / Unknown |
| C.17 | What kind of the composite materials (in large quantity) are still/was in place? | Multiple Choice: Single Answer | Fibre reinforced polymer / Ceramic / Steel reinforced concrete / Composite wood beam / Others |
| C.18 | What percentage of the total building component can be/was reused? | Multiple Choice: Single Answer | < 25% / 25-50% / 50-99% / 100% |
| C.19 | What percentage of the total building component can be/was recycled? | Multiple Choice: Single Answer | < 25% / 25-50% / 50-99% / 100% |
| C.20 | What percentage of the total building component will be/was sent to landfill? | Multiple Choice: Single Answer | < 25% / 25-50% / 50-99% / 100% |
| **Section D** | | | |
| D.1 | Was/is there a fireplace mantel | Multiple Choice: Single Answer | Yes / No / Unknown |
| D.2 | Was/is there a stair treads/railing | Multiple Choice: Single Answer | Yes / No / Unknown |
| D.3 | Was/is there other architectural woodworks | Multiple Choice: Single Answer | Yes / No / Unknown |
| D.4 | Was/is there stained/leaded glass | Multiple Choice: Single Answer | Yes / No / Unknown |
| D.5 | Was/is there solid wood doors | Multiple Choice: Single Answer | Yes / No / Unknown |
| D.6 | Was/is there wood framed windows | Multiple Choice: Single Answer | Yes / No / Unknown |
| D.7 | Was/is there built-in wood cabinetry | Multiple Choice: Single Answer | Yes / No / Unknown |
| D.8 | Was/is there decorative architectural wrought iron | Multiple Choice: Single Answer | Yes / No / Unknown |
| D.9 | Was/is there lighting fixtures | Multiple Choice: Single Answer | Yes / No / Unknown |
| D.10 | Was/is/are there radiators | Multiple Choice: Single Answer | Yes / No / Unknown |
| D.11 | Was/is/are there sinks | Multiple Choice: Single Answer | Yes / No / Unknown |
| D.12 | Was/is/are there claw foot tub | Multiple Choice: Single Answer | Yes / No / Unknown |
| D.13 | Was/are there old appliances (oven, refrigerator) | Multiple Choice: Single Answer | Yes / No / Unknown |
| D.14 | Was/is there iron gates/fencing | Multiple Choice: Single Answer | Yes / No / Unknown |
| D.15 | Was/is there metal roofing | Multiple Choice: Single Answer | Yes / No / Unknown |
| D.16 | Was/are there countertops | Multiple Choice: Single Answer | Yes / No / Unknown |
| D.17 | Was/is there door hardware | Multiple Choice: Single Answer | Yes / No / Unknown |
| D.18 | Was/are there other old/rare steels | Multiple Choice: Single Answer | Yes / No / Unknown |
| D.19 | ﻿Are you aware of any architectural salvage yards? | Multiple Choice: Single Answer | Yes / No / Unknown |
| D.20 | Were there any other architectural components present in the building with historic/commercial value? | Multiple Choice: Single Answer | Yes / No / Unknown |
| **Section E** | | | |
| E.1 | What was/is the percent of Claddings reusable/recyclable? | Multiple Choice: Single Answer | 1 (little) / 2 / 3 / 4 / 5 (lots) |
| E.2 | What was/is the percent of Connections reusable/recyclable? | Multiple Choice: Single Answer | 1 (little) / 2 / 3 / 4 / 5 (lots) |
| E.3 | What was/is the percent of Frameworks reusable/recyclable? | Multiple Choice: Single Answer | 1 (little) / 2 / 3 / 4 / 5 (lots) |
| E.4 | What was/is the percent of Glazing reusable/recyclable? | Multiple Choice: Single Answer | 1 (little) / 2 / 3 / 4 / 5 (lots) |
| E.5 | What was/is the percent of Insulation reusable/recyclable? | Multiple Choice: Single Answer | 1 (little) / 2 / 3 / 4 / 5 (lots) |
| E.6 | How expensive is/was it to hire deconstruction worker in your region | Multiple Choice: Single Answer | Cheap / Expensive / Not sure |
| E.7 | How expensive is/was the tipping/disposal fee for dumping waste in landfill in your region? | Multiple Choice: Single Answer | Cheap / Expensive / Not sure |
| E.8 | How expensive is/was it to get deconstruction/disassembly permit from the government/local authority? | Multiple Choice: Single Answer | Cheap / Expensive / Not sure |
| E.9 | How expensive is/was it to get a trained toxic/hazardous material handler in your region? | Multiple Choice: Single Answer | Cheap / Expensive / Not sure |
| E.10 | How expensive is/was it to get specialised equipment/tools? | Multiple Choice: Single Answer | Cheap / Expensive / Not sure |
| E.11 | ﻿Are you aware of any stockists of reclaimed components or elements? | Multiple Choice: Single Answer | Yes / No |
| E.12 | Are there any restrictions or limitations on the availability of certain materials due to environmental regulations or building codes? | Multiple Choice: Single Answer | Yes / No |
| E.13 | Would you consider reclaiming components from a project if you knew there was good demand for them, and they were commercially viable? | Multiple Choice: Single Answer | Yes / No |
| E.14 | ﻿Are you in the position to supply spare parts or to provide a reconditioning service on demand or to undertake reconditioning as a core service? | Multiple Choice: Single Answer | Yes / No |
| E.15 | Were tests available to assess the condition/life expectancy of materials (both in-situ and ex-situ)? | Multiple Choice: Single Answer | Yes / No |
| **Section F** | | | |
| F.1 | Which stakeholders would you say have the significant influence on your deconstruction activities | Multiple Choice: Single Answer | Building owner / Contractor / Client / Government / Others |
| F.2 | ﻿What would you say was/is the main motivation for the deconstruction? | Multiple Choice: Single Answer | Sustainability badge / Economic gain / Job creation / Social responsibility / Others |
| F.3 | Does the government policy encourage deconstruction in the region? | Multiple Choice: Single Answer | Yes / No |
| F.4 | Does the public attitude encourage deconstruction in the region? | Multiple Choice: Single Answer | Yes / No |
| F.5 | ﻿Do you believe that the benefits of material reuse are well-understood by the public? | Multiple Choice: Single Answer | Yes / No / Not sure |
| **Section G** | | | |
| G.1 | ﻿What was/is the estimated time it took or the estimated time it will take to for jobsite preparation? | Multiple Choice: Single Answer | Few hours / A Day / Few days / Weeks / Others |
| G.2 | What was/is the estimated time it took or the estimated time it will take to get permit to deconstruction permit? | Multiple Choice: Single Answer | Few hours / A Day / Few days / Weeks / Others |
| G.3 | What was/is the estimated time it took or the estimated time it will take to deconstruct the building? | Multiple Choice: Single Answer | Few hours / Days / Weeks / Months / Others |
| G.4 | What was/is the estimated time it took or the estimated time it will take to assess the building for deconstruction? | Multiple Choice: Single Answer | Few hours / A Day / Few days / Weeks / Others |
| G.5 | What was/is the estimated time it took or the estimated time it will take to sort the recovered building components? | Multiple Choice: Single Answer | Few hours / Days / Weeks / Months / Others |
| G.6 | What time of the year was/is the disassembly/deconstruction (done or proposed to be done)? | Multiple Choice: Single Answer | Rainy / Non-Rainy / Not sure |
| G.7 | ﻿We would like to keep in touch with you about this  survey. If you would be willing to be contacted, please  provide contact information (Phone number or email) | Free Text |  |

## Appendix A2: **Mapping Questionnaire Variables to Deconstructability Dimensions**

*Table A2.* ***Mapping Questionnaire Variables to Deconstructability Dimensions***

| **Deconstructability Dimension** | **Mapped Variables** | **Description** |
| --- | --- | --- |
| **Technical** (Building design, material composition, modularity, structural accessibility, construction methods) | **B.3** Construction method, **B.16** Accessibility of structural connections (also Scheduling), **B.18** Roof type, **B.19** Foundation type, **B.29** Availability of building design/inventory (also Legal), **B.30** Disassembly plan (also Scheduling & Economic), **C.1 - C.20** Material composition & recoverability (also Environmental & Economic), **D.1 - D.20** Architectural salvage components (also Economic & Environmental) | Evaluates how the structure’s design, materials, and modularity affect deconstructability and material recovery. |
| **Economic** (Cost feasibility, material resale value, labor, and disposal costs) | **E.6** Deconstruction labor costs, **E.7** Landfill disposal fees, **E.8** Government permit costs (also Legal), **E.9** Toxic material handling costs (also Environmental & Legal), **E.10** Equipment/tool costs, **E.11** Availability of reclaimed material suppliers (also Social), **E.13** Viability of salvaged materials for resale (also Environmental), **E.14** Reconditioning services, **B.30** Disassembly plan (also Technical & Scheduling) | Assesses financial factors influencing the feasibility of deconstruction, including labour, disposal, and material resale. |
| **Social** (Stakeholder roles, public perception, workforce impact) | **F.1** Stakeholder influence, **F.2** Motivation for deconstruction (also Economic), **F.3** Government policy support (also Legal & Economic), **F.4** Public attitude, **F.5** Public awareness of reuse benefits, **E.11** Availability of reclaimed material suppliers (also Economic) | Captures societal influences, including public and industry attitudes toward deconstruction and reuse. |
| **Environmental** (Sustainability, waste reduction, hazardous materials, ecological concerns) | **B.14** On-site hazards (also Legal & Scheduling), **B.20** Proximity to recycling facilities (also Economic), **B.21 - B.26** Structural deterioration indicators, **B.27** Types of hazardous materials identified (also Legal), **B.28** Quantity of hazardous materials, **C.1 - C.20** Material composition & recoverability (also Technical & Economic), **D.1 - D.20** Architectural salvage components (also Economic & Technical), **E.12** Environmental regulations affecting material reuse (also Legal) | Focuses on sustainability aspects, including material recovery potential, waste reduction, and hazardous material considerations. |
| **Legal** (Regulatory compliance, safety protocols, permitting processes) | **E.8** Cost of obtaining a deconstruction permit (also Economic), **E.12** Regulatory restrictions on material reuse (also Environmental), **B.29** Availability of building design/inventory (also Technical), **B.27** Types of hazardous materials identified (also Environmental), **B.14** On-site hazards (also Environmental & Scheduling), **E.9** Cost of hazardous material handling (also Economic & Environmental), **F.3** Government policy support (also Social & Economic) | Examines regulatory and safety requirements affecting deconstruction and material reuse. |
| **Scheduling** (Project timelines, site constraints, logistical feasibility) | **B.9** Site security against unauthorized entry (also Legal), **B.10** Availability of staging area (also Technical), **B.11 - B.13** Presence of exterior/interior waste and movement restrictions (also Environmental & Legal), **B.16** Accessibility of structural connections (also Technical), **B.30** Disassembly plan (also Technical & Economic), **G.1 - G.6** Estimated time for preparation, permitting, deconstruction, material sorting, and seasonal constraints | Evaluates logistical and time-based challenges associated with site preparation and deconstruction execution. |
